# Allosteric Modulation of Beta1 Integrin Function Induces Lung Tissue Repair

**DOI:** 10.1155/2012/768720

**Published:** 2012-02-26

**Authors:** Rehab AlJamal-Naylor, Linda Wilson, Susan McIntyre, Fiona Rossi, Beth Harrison, Mark Marsden, David J. Harrison

**Affiliations:** ^1^Avipero Ltd., 5th Floor, 125 Princes Street, Edinburgh EH2 4AD, UK; ^2^School of Biomedical Sciences, The University of Edinburgh, Hugh Robson Building, George Square, Edinburgh EH8 9XD, UK; ^3^Division of Pathology, Institute of Genetics and Molecular Medicine, The University of Edinburgh, Western General Hospital, Edinburgh EH4 2XU, UK; ^4^MRC Centre for Inflammation Research, The Queen's Medical Research Institute, The University of Edinburgh, 47 Little France Crescent, Edinburgh EH16 4TJ, UK

## Abstract

The cellular cytoskeleton, adhesion receptors, extracellular matrix composition, and their spatial distribution are together fundamental in a cell's balanced mechanical sensing of its environment. We show that, in lung injury, extracellular matrix-integrin interactions are altered and this leads to signalling alteration and mechanical missensing. The missensing, secondary to matrix alteration and cell surface receptor alterations, leads to increased cellular stiffness, injury, and death. We have identified a monoclonal antibody against *β*1 integrin which caused matrix remodelling and enhancement of cell survival. The antibody acts as an allosteric dual agonist/antagonist modulator of *β*1 integrin. Intriguingly, this antibody reversed both functional and structural tissue injury in an animal model of degenerative disease in lung.

## 1. Introduction

Tissue regeneration comprises dedifferentiation of adult cells into a stem cell state and the development of these cells into new remodelled tissue, identical to the lost one. Tissue repair is defined as replacement of normal tissue by fibrous tissue and integrins are crucial in these processes.

Integrins are membrane spanning proteins facilitating the two-way communication between the inside and outside of a cell. Integrins have the capacity to bind a multitude of molecules both inside and outside of the cell. The binding of these molecules results in the transmission of information into and out of the cell, which can influence a host of different cellular functions, including the cells metabolic activity.

Of the many types of integrin receptors, the *β*1 integrin is by far the most ubiquitous allowing cells to detect a vast array of stimuli ranging between toxins, protein hormones, neurotransmitters, and macromolecules. There have been numerous publications documenting a potential role of *β*1 integrin in tissue development and repair in several tissue types (reviewed in [1]). It is clear that *β*1 integrin plays a crucial role during postnatal skin development and wound healing, with the loss of epithelial *β*1 integrin causing extensive skin blistering and wound healing defects. More recently, there has been active interest in the cosmeceutical development of *β*1 integrin targeting formulations. One such example is following the discovery of fucoidans from* Fucus vesiculosus* and its effect on skin scarring and ageing [2, 3] which was later found to be mainly attributed to alpha2 and *β*1 integrin [4].

Integrins in general, including *β*1 integrin, exhibit global structural rearrangement and exposure of ligand binding sites upon activation [5]. The overall strength of cellular adhesiveness, or avidity, is governed by affinity and valency (which is in turn governed by the density of the receptor and its ligand on the cell surface, as well as the spatial and geometric arrangement and movement) [5]. Recent evidence has demonstrated that both affinity and avidity of integrins are strongly related to the size of the focal adhesion clusters [6, 7]. Overall, integrins have three main possible conformations of the extracellular domain; a low affinity, bent conformation; extended conformation with closed headpiece representing an intermediate affinity state; the ligand-binding-induced high-affinity extended form, with an open headpiece [8–10]. 

Altered conformation of integrins rather than expression levels have been reported both during physiological and pathological remodelling processes which include neurite outgrowth, fibrosis, asthma, cancer, and wound healing amongst many [11–14]. To successfully develop disease modifying therapy, it should be beneficial to rescue or replenish dying or dead cells by activating inherent repair processes other than simply stem cell regeneration. In other words, altering the interaction of the cells with other cells and their abnormal surroundings to promote their survival and continued function may alleviate chronic, ongoing cell loss, a hallmark of many progressive degenerative diseases. We hypothesised that tissue repair might be achieved by rescueing cells from death by mechanically dampening the signals cells receive from their abnormal environment. One key cell surface receptor for adhesion is *β*1 integrin. We considered that conformational modulation of *β*1 integrin may cause alteration in cytoskeletal organization and cell stiffness leading to increase susceptibility to oxidative stress and death and thus that prevention of these changes may have therapeutic benefit. Here, we show how we have identified a specific *β*1 integrin targeting modality using a monoclonal antibody, which we have demonstrated, both protects from tissue injury and facilitates repair. The antibody acts as an allosteric dual agonist/antagonist modulator of *β*1 integrin and resulted in increased matrix remodelling and enhancement of cell survival. 

## 2. Results

### 2.1. The Effects of *β*1 Integrin Modulation on Elastase-Induced Signalling

As a model of tissue remodelling in disease, we investigated the activity of *β*1 integrin using an *in vitro* model of elastase-induced injury. A coculture of primary, adult human lung fibroblasts was overlayered with NCI-H441 lung cells, under cyclic mechanical stimulation, and subjected to elastase treatment.

To investigate the involvement of *β*1 integrin activation in elastase-induced signalling, we used three different monoclonal antibodies against *β*1 integrin. The first was the adhesion blocking clone, JB1a, which is said to target primarily the amino acids 82–87 comprising part of the hybrid domain [15]. We also used the adhesion blocking clone, AIIB2, which binds amino acid residues 207–218 within the A-domain [16], and K20, widely reported to have no functional effects which binds the hybrid/EGF repeat region [17].

**Figure 1:**
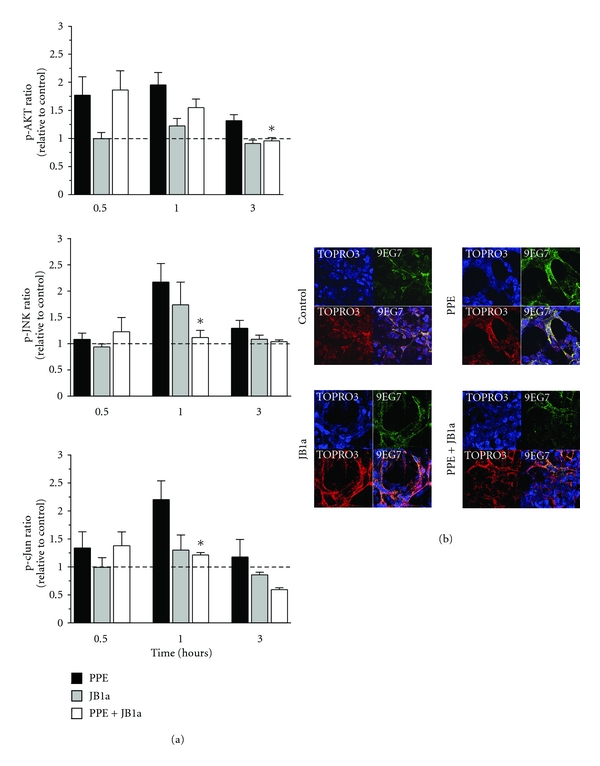
The effects of PPE-induced injury and JB1a treatment on (a) activation of signalling downstream of beta1 integrin during mechanical stretch (asterisks denote statistical significance with *P* < 0.05 in comparison to PPE) and (b) beta1 integrin conformational activation indicated by increase in staining using the anti-*β*1 integrin antibody 9EG7 recognising the ligand competent receptor in comparison to staining using nonconformation dependent antibody (K20).

Addition of elastase to cell culture induced an increased phosphorylation of signalling proteins known to act downstream of *β*1 integrin (Figure 1(a)). During the course of injury, pAKT levels increased, followed by transient increases in phospho-cJUN and phospho-JNK (Figure 1(a)). No significant changes were detected for 12 other phosphoproteins at the sampled time points.

**Figure 2:**
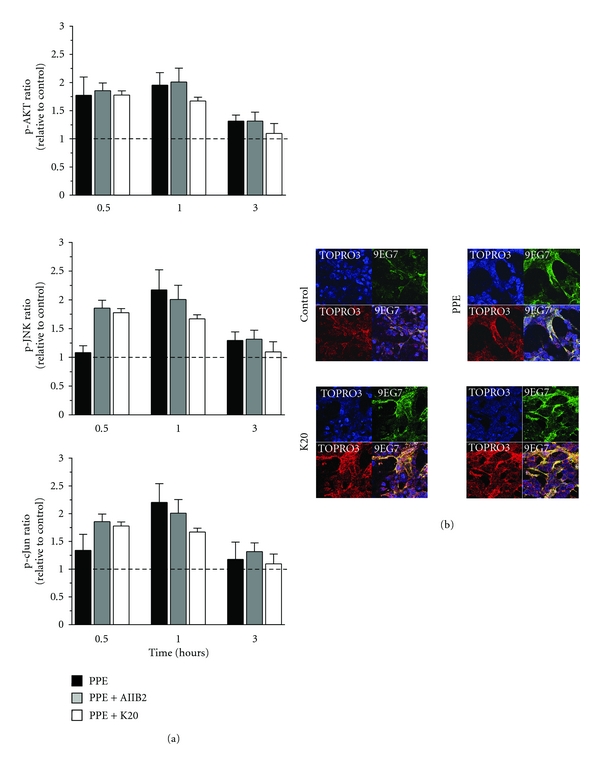
The effects of PPE-induced injury and targeting beta1 integrin using AIIB2 and K20 clones on (a) activation of signalling downstream of beta1 integrin during mechanical stretch (asterisks denotes statistical significance with *P* < 0.05 in comparison to PPE) and (b) beta1 integrin conformational activation indicated by increase in staining using the anti-*β*1 integrin antibody 9EG7 recognising the ligand competent receptor in comparison to staining using nonconformation dependent antibody (K20).

When *β*1 integrin was bound by antibody clone JB1a in the absence of injury, there was no significant effect on downstream signalling. However, when JB1a was added during elastase-induced injury, it abrogated all elastase-induced changes in phosphorylation of signalling proteins. This effect was not seen with either AIIB2 or K20 (Figure 2(a)).

### 2.2. The Effects of Elastase-Induced Injury on *β*1 Integrin Activity and Localisation

We next examined the effects of elastase on ligand-binding activity of *β*1 integrin using the ligand competent specific anti-*β*1 integrin antibody 9EG7. Ligand competent state can be any of the intermediate physiological conformations or the fully activated extended conformation [19]. Elastase caused an increase in ligand competent/active *β*1 integrin expression as evident from the staining pattern in Figure 1(b). Modulation of *β*1 integrin, using JB1a, abrogated the elastase-induced increase in ligand-competent *β*1 integrin (Figure 1(b)). However, the anti-*β*1 integrin clone K20 potentiated the elastase-induced increase in the ligand-competent conformation (Figure 2(b)).

**Figure 3:**
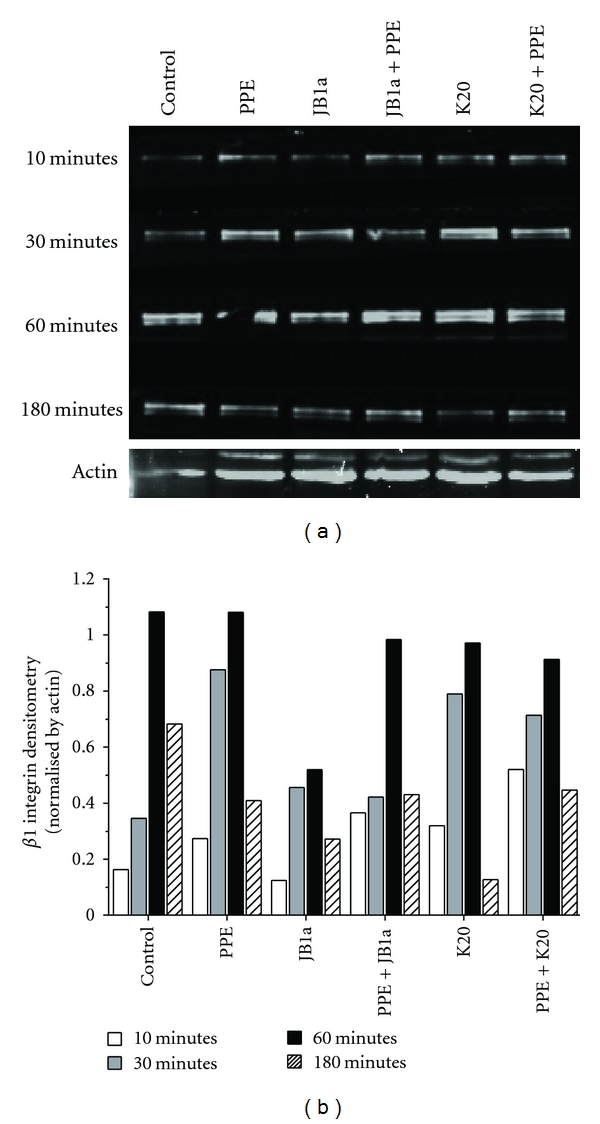
The effects of PPE-induced injury (0.6 U/mL) and targeting beta1 integrin using JB1a (1 ug/mL) in comparison to K20 (1 ug/mL) clone on the kinetics of beta1 integrin levels on cell membrane *in vitro* using human lung coculture. (a) Western blot of the cell membrane expression of *β*1 integrin over time. Protein extracts were loaded at equal protein concentration (25 *μ*g). (b) Densitometric analyses of the blot corrected using actin as an internal control.

**Figure 4:**
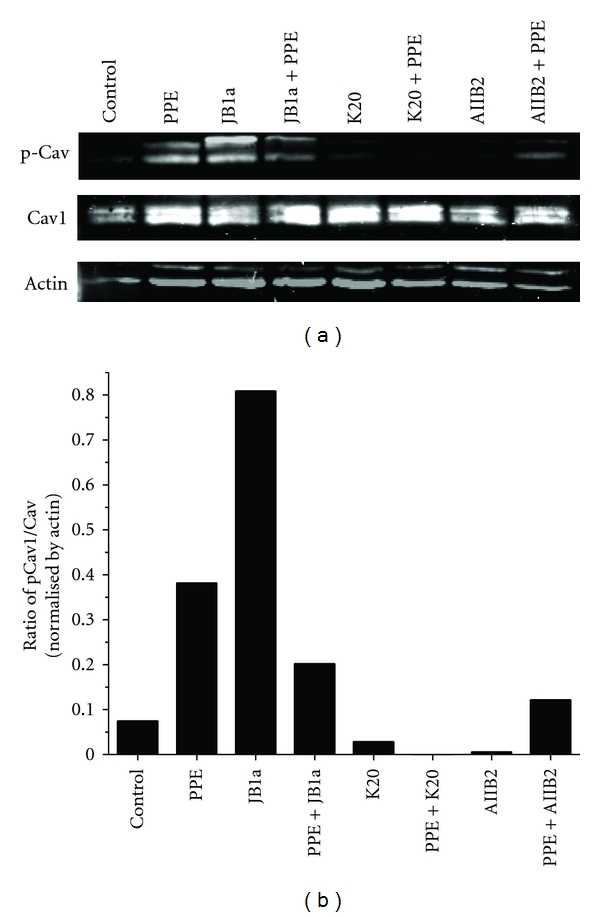
The effects of PPE-induced injury (0.6 U/mL) and targeting beta1 integrin using AIIB2 (1 ug/mL) and K20 (1 ug/mL) clones on phosphorylated caveolin-1 levels in membrane fractions. (a) Representative blots from *n* = 4. Loading controlled by total amount of protein (50 *μ*g). (b) Densitometric analyses of the blot corrected using actin as an internal control.

The effects of elastase and JB1a on the level of ligand-competent receptor were not simply a result of change in cell surface expression (Figure 1(c)). However, preliminary measurements showed that elastase increased the cytosolic fraction-associated *β*1 integrin which might be attributed to recycling or degradation. To address that we conducted a time course analyses of *β*1 integrin in membrane fractions. Elastase induced a change in *β*1 integrin recycling, an effect inhibited by JB1a but not K20 (Figure 3). Further evidence of elastase-induced *β*1 integrin activation was the increase of caveolin-1 phosphorylation after two hours of exposure to elastase; changes once again inhibited by JB1a (Figure 4). 

### 2.3. The Conformational Effects of JB1a on *β*1 Integrin

**Figure 5:**
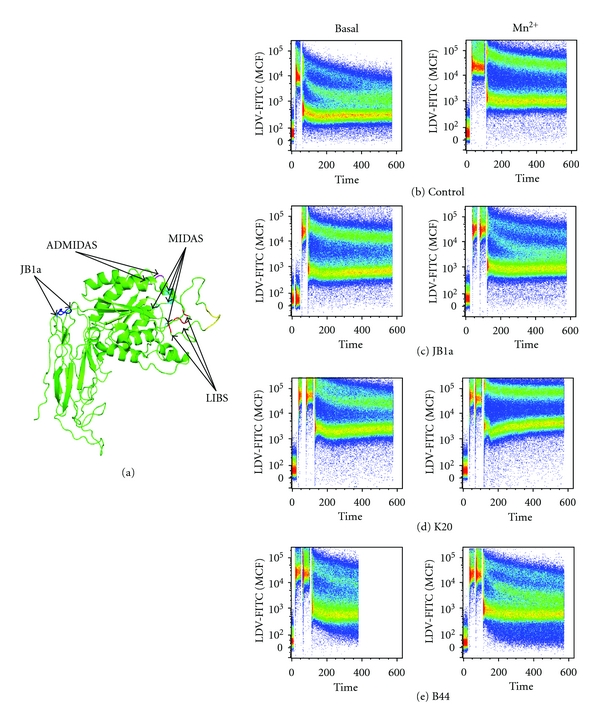
(a) The location of JB1a epitope as mapped by Ni and Wilkins [15] produced using polyview 3D as described in [18] http://polyview.cchmc.org/polyview3d.html FRET analyses demonstrating. (b) the baseline conformation of beta1 integrin and following Mn^2+^-induced integrin activation. The effect of JB1a (c), K20 (d), and B44 (e) on integrin at baseline and on Mn^2+^-induced integrin activation detected by FRET using the LDV-FITC small molecule and R18 in Jurkat cells. LDV binding is plotted as mean channel fluorescence (MCF) versus time.

To further determine the pharmacological mode of action of JB1a action, we questioned whether the effect seen with JB1a is due to its effect on *β*1 integrin chain allostery. We first estimated the location of the epitope of JB1a on the basis of the theoretical 3-dimentional structure of *β*1 integrin (Figure 5(a)). We then conducted FRET studies using nonadherent Jurkat cells. FITC-labelled LDV cyclic peptide was used to label the head of alpha4*β*1 integrin, and the lipophilic dye R18 was used to label the cell membrane. LDV-FITC acts as a donor and R18 as an acceptor [20]. FACS was used for the FRET acquisition and measurement. JB1a caused a conformational activation when added at baseline and inhibited the full conformational activation induced by the divalent cation, Mn^2+^. The JB1a-induced change resulting in FRET efficiency was indicative of an intermediate partially extended conformation when compared to Mn^2+^ and other known inhibitory or activating antibodies (Figures 5(b)–5(e)). 

Taking together these findings indicated that the pharmacological mode of action of JB1a-mediated effect on *β*1 integrin is as an allosteric dual agonist/antagonist.

### 2.4. Conformational Modulation of *β*1 Integrin Inhibits Elastase-Induced Changes in Cell Membrane Composition

**Figure 6:**
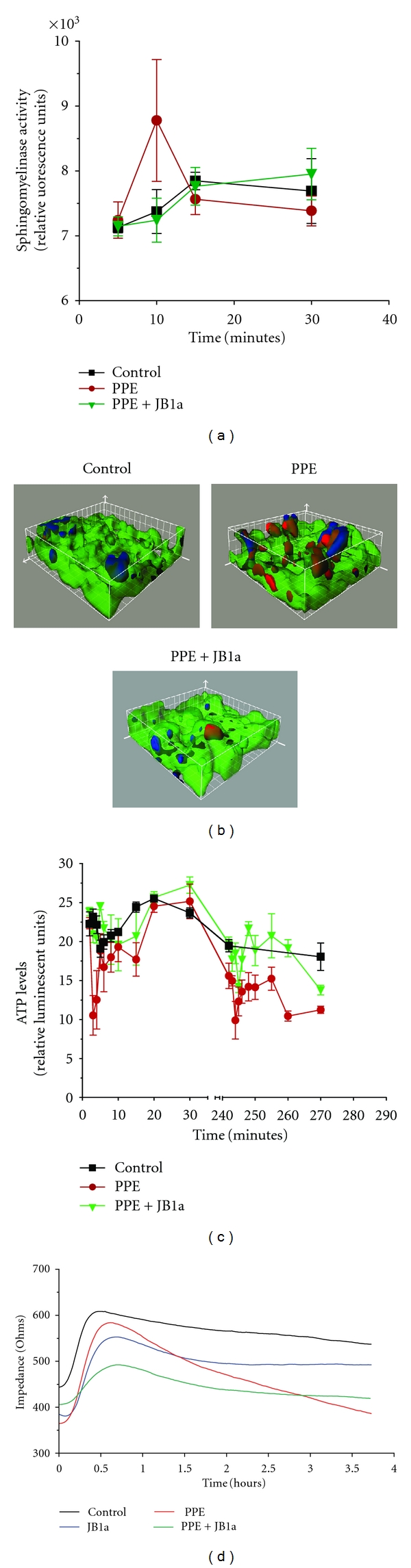
The effects of PPE-induced injury (0.6 U/mL) and JB1a treatment (1 ug/mL) *in vitro* using human lung coculture cultured on collagen-coated surfaces. The effects measured were on (a) neutral sphingomyelinase activity one on cultures subjected to mechanical stretch of 2–10% amplitude at 1 Hz (**n** = 3), (b) F-actin using 3D reconstruction of images of human lung coculture after injury using elastase demonstrating the formation of F-actin (blue) and caspase 3/7 activation (red). Ganglioside GM1 for the cell membrane-green and its inhibition by JB1a done on cells cultured on glass (**n** = 3), (c) ATP levels (**n** = 3 and each included separate measurements of cells cultured in 8 wells in 96-well plates). (d) Cellular electrical impedance (**n** = 3).

Using mixed epithelial-mesenchymal *in vitro* cultures, we found that elastase increased neutral sphingomyelinase activity transiently; an effect inhibited by *β*1 integrin binding by antibody JB1a (Figure 6(a)). No effect on acid sphingomyelinase was detected under the same conditions. 

### 2.5. Conformational Modulation of *β*1 Integrin Inhibits Elastase-Induced Changes in Actin Polymerisation and Cellular Impedance

Using our *in vitro* culture system, we tracked incorporation of labelled monomeric actin, and demonstrated an increase in de novo F-actin formation during the course of elastase-induced injury (Figures 6(b), 12, and S1–S3 in Supplementary Material available online at doi:10.1155/2012/768720). Formation of F-actin from monomeric G-actin is energy dependent, and, under ATP depletion conditions, there is a net conversion of monomeric G-actin to polymeric F-actin. In cocultures, elastase reduced the levels of ATP, but this response was inhibited by JB1a (Figure 6(c)).

To corroborate the finding on cellular mechanical properties, we investigated the effect of elastase on cellular impedance. There was an initial drop and recovery in impedance after change of media consistent with responses to sudden stretch, as reported previously [21]. JB1a inhibited the elastase-induced decrease in cellular impedance (Figure 6(d)).

### 2.6. Conformational Modulation of *β*1 Integrin Inhibits Elastase-Induced Caspase Activation

**Figure 7:**
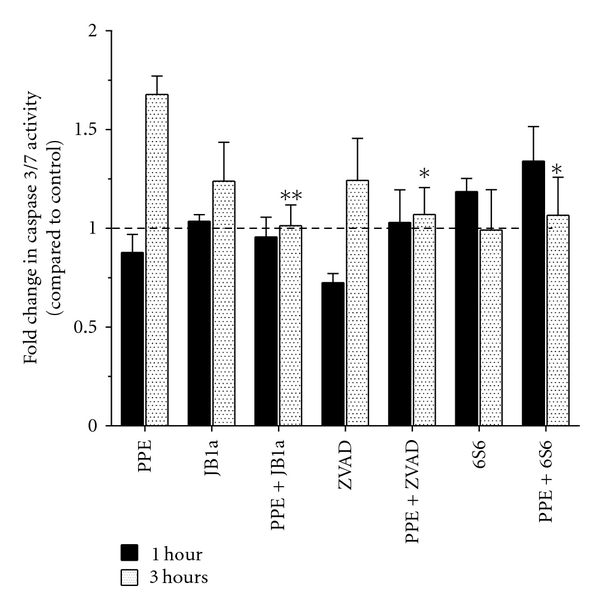
The effects of PPE-induced injury (0.6 U/mL) and targeting beta1 integrin using JB1a (1 ug/mL) in comparison to 6S6 clone (1 ug/mL) and the broad spectrum caspase inhibitor, ZVAD-fmk, on caspase 3/7 activation *in vitro* using human lung coculture during mechanical stretch (*n* = 3). Asterisks denotes statistical significance with *P < 0.05 and **P < 0.005 in comparison to PPE.

We then investigated the effect of elastase on caspase activation and the role of *β*1 integrin in elastase-induced cell death. Elastase induced caspase activation after 3-hour exposure and led to detachment-induced apoptosis of epithelial cells (anoikis) (Figures 7(a) and S1–S3). Modulation of *β*1 integrin using JB1a prevented caspase activation. However, the potently inhibitory anti-*β*1 integrin antibody 6S6, which is also known to induce homotypic aggregation, induced caspase activation (Figure 7(b)).

### 2.7. Conformational Modulation of *β*1 Integrin Reversed Elastase-Induced Emphysema in Mice

**Figure 8:**
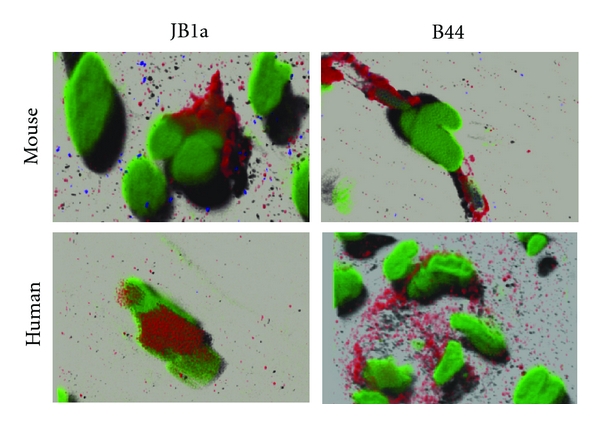
JB1a and B44 immunoreactivity with beta1 integrin verification on human tissues and their cross-reactivity with beta1 integrin in mouse tissue. Images were collected using ×40 oil lens and Zeiss LSM510 CLSM microscope with nyquist settings. The resulting images were deconvolved, and three-dimensional images were reconstructed using Huygens software (Scientific Volume Imaging (SVI), The Netherlands).

To investigate the significance of *β*1 integrin in injury in a disease setting in which remodelling is a key component, we established a murine model of emphysema caused by intratracheal installation of elastase. Mice were instilled with elastase on day 1 and lung injury ensued. At later timepoints, they were treated with the anti-*β*1 integrin monoclonal antibody, JB1a which binds *β*1 integrin in mouse tissues [22], or vehicle, either once on day 14 (21 day group, 21 d) or on days 21 and 28 (35 day group, 35 d). In a subsequent investigation, severe emphysema was induced and JB1a and B44 clones were instilled on days 21 and 28 before lung function assessment on day 35. Both clones demonstrated cross-reactivity with murine *β*1 integrin (Figure 8).

**Figure 9:**
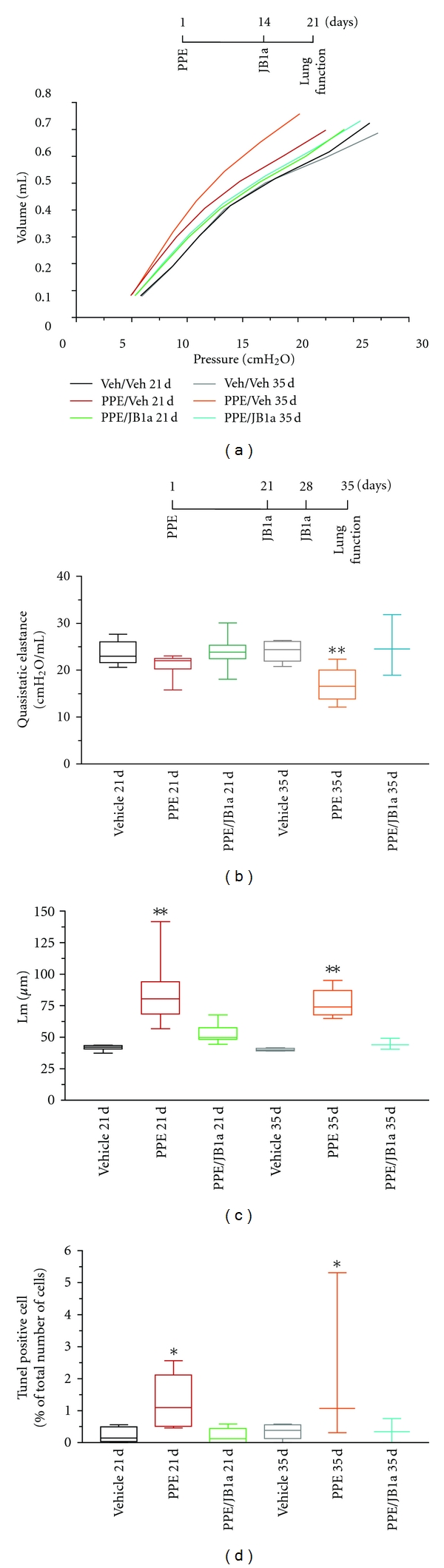
The effect of porcine pancreatic elastase (PPE, 0.2 U/g) on respiratory function in mice and its reversal using the anti-beta1 integrin antibody JB1a (3 mg/kg). (a) The effect of PPE on mean respiratory pressure-volume curves in mice from the 21days (21 d) and 35 days (35 d) after instillation and its reversal by JB1a (vehicle = Veh). (b) Reversal of PPE-induced increase in the quasistatic elastance between 5 and 9 cm H_2_O by JB1a treatment at different time points after injury. (c) Mean linear intercept (Lm) measurements from the 21 d and 35 d groups. *n* = 5-6 in 35 d groups and *n* = 10 in 21 d groups. (d) TUNEL staining demonstrating the effect of JB1a treatment after PPE-induced lung injury. (c) quantification of TUNEL positive cells in lung tissue sections from 21 d and 35 d group following PPE-induced injury and JB1a treatment (*n* = 5-6 per group). Asterisks denote statistical significance with *P < 0.05, **P < 0.005 and ***P < 0.0005 in comparison to vehicle.

By 21 days after elastase injury, there was a marked progressive leftward shift in the respiratory pressure-volume curve (PV) of close-chested mice, particularly in the 35 day group (Figure 9(a)). JB1a, given as a single intratracheal dose at this time point, reversed the loss of respiratory elastic recoil induced by elastase treatment (Figure 9(b)).

**Figure 10:**
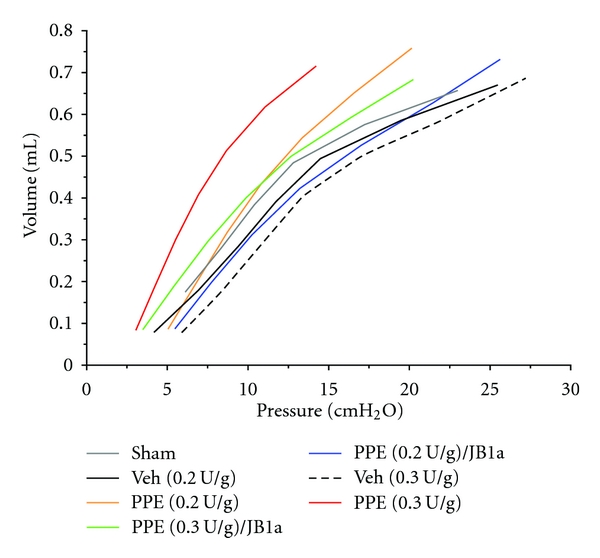
Dose-response of the effects of porcine pancreatic elastase (PPE, 0.2 U/g and 0.3 U/g) on respiratory function in mice and its reversal using the anti-beta1 integrin antibody JB1a. The effect of PPE on mean respiratory pressure-volume curves in mice from 35 days (35 d) after instillation and its reversal by JB1a (vehicle = Veh).

In addition to the reversal of functional characteristics, treatment with JB1a was associated by structural repair, assessed by histology and morphometry (Figure 9(c)). In elastase-treated lungs, apoptosis was demonstrated by the TUNEL assay at 21 and 35 days, even in the absence of inflammation. This was prevented by JB1a treatment (Figure 9(d)). There was no change in cellular proliferation as assessed by immunostaining for Ki67. The efficacy of *β*1 integrin modulation using the clone JB1a was evident even in more severe injury when a higher dose of elastase was used. (Figure 10).

**Figure 11:**
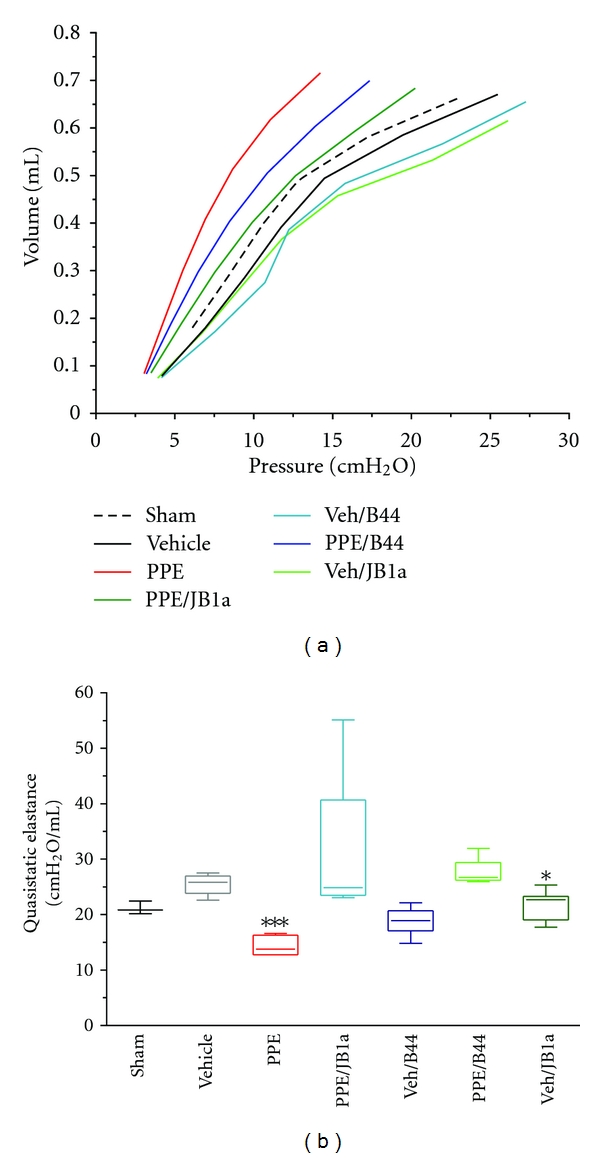
The effects of porcine pancreatic elastase (PPE, 0.3 U/g) on respiratory function in mice and its reversal using the anti-beta1 integrin antibody JB1a (3 mg/kg) in comparison to the anti-beta1 integrin clone B44 (3 mg/kg). (a) The effect of PPE on mean respiratory pressure-volume curves in mice from 35 days (35 d) after instillation and its reversal by JB1a and not B44. (b) Reversal of PPE-induced increase in the quasi-static elastance between 5 and 9 cm H_2_O by JB1a treatment (**n** = 6–10). Asterisks denote statistical significance with *P < 0.05, **P < 0.005, and ***P < 0.0005 in comparison to vehicle.

**Figure 12:**
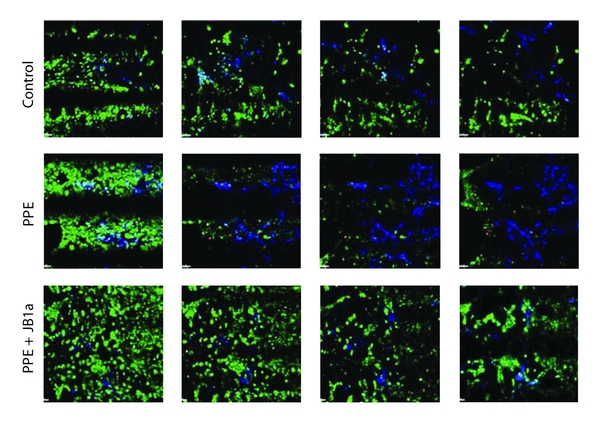
Selected frames from time lapse videos of epithelial-mesenchymal cultures during stretch (compressed videos) demonstrating the formation of F-actin (blue) and caspase 3/7 activation (red) in reponse to elastase (PPE, 0.6 U/mL) and its inhibition by JB1a done on cells cultured on glass. Sytox green was used for cell tracking. (a) control, (b) PPE (0.6 U/mL), and (c) PPE + JB1a (1 ug/mL).

When we tested the effect of *β*1 integrin modulation using JB1a in comparison to the clone B44 following the same protocol of the 35 day group, B44 had no significant effect at a comparable dose (Figures 11(a) and 11(b)). The clone B44 bears the closest resemblance in its conformational effect to the JB1a from our FRET results. In parallel studies, we tested the potent inhibitory antibody, 6S6 known to induce homotypic aggregation. Whilst 6S6 had no effect in control animals, its effect on elastase-treated animals was detrimental and worsened injury corroborating it proapoptotic effect* in vitro*.

## 3. Discussion

In this paper we have investigated the role of *β*1 integrin in lung injury and repair in emphysema. We demonstrated that *β*1 integrin becomes allosterically activated in epithelial-mesenchymal cells, with the corollary that allosteric modulation inhibited elastase-induced injury. We further demonstrate a potential cellular mechanism for this *β*1 integrin-mediated effect. In order to do so, we established an *in vitro* model system which replicated features of elastase-induced emphysema *in vivo*. We identified that allosteric modulation of *β*1 integrin inhibited caspase activation, F-actin aggregate formation, and abnormal fluctuations in cellular ATP levels, under conditions in which the total *β*1 expression was changed and activation inhibited. The key finding of our investigation was that, by direct allosteric modulation of *β*1 integrin with a specific monoclonal antibody, both functional and structural reversal of elastase-induced tissue injury was induced *in vivo*. Our findings support the notion that cytomechanics are important determinants of cell fate and effect repair.

Upon activation, integrin-linked kinase (ILK) binds to the cytoplasmic domain of the *β*1 integrin subunit [23]. In turn, ILK activates multiples signalling pathways such as protein kinase B (PKB/AKT) and inhibits glycogen synthase kinase-3*β* (GSK-3*β*) activity affecting transcription factor binding to their DNA sequences [23–25]. We demonstrated that elastase-induced injury activated signalling downstream of *β*1 integrin and this effect was modulated by targeting *β*1 integrin using the clone JB1a. Although JB1a is known as an inhibitory antibody, the effect on elastase-induced signalling was specific to JB1a since targeting *β*1 integrin using the inhibitory clone AIIB2 did not have the same effect nor did the clone K20. The elastase-induced activation of *β*1 integrin was corroborated by demonstrating that there was an increased detection of ligand-competent *β*1 integrin which was not caused by increased protein level but rather increased recycling. By contrast, the anti-*β*1 integrin clone K20 induced an increase in the ligand-competent conformation; an effect previously noted [26].

The separation of the alpha and *β* subunit legs is a critical step in integrin activation to transform the bent structure to an extended conformation, thus allowing headpiece-ligand engagement [8]. Therefore, we questioned whether the effect seen with JB1a is due to its effect on *β*1 integrin chain allostery. Indeed, targeting amino acid sequences within the same epitope of JB1a in the hybrid domain region using other antibodies has been reported to stabilise the physiological intermediate state of the receptor in a similar fashion as an allosteric antagonist [8]. We adopted an assay method used to detect conformational changes in integrin [20, 27] and found that, under baseline conditions, JB1a had an activating effect whilst it acted as a conformational antagonist when *β*1 integrin was activated with manganese. We have examined 8 other clones and determined that the clones closest to JB1a in its conformational effect were the B44 and HUTS 21 clones; both of which bind to the second hybrid domain of *β*1 integrin (reviewed in [1]). Therefore, adding to the reported effects of JB1a, we have shown that it functions both as an agonist and antagonist.

We then sought to elucidate the significance of integrin activation in response injury. Receptor clustering is, in part, aided by interactions with cellular proteins such as caveolins cell membrane fluidity. The composition of cell plasma membrane directly affects *β*1 integrin function and membrane fluidity in response to other types of injury [28, 29], reviewed in [1]. In using *in vitro* mixed epithelial-mesenchymal cultures, we found that elastase increased neutral sphingomyelinase activity transiently; an effect inhibited by *β*1 integrin binding by antibody JB1a. The association of neutral sphingomyelinase has been shown recently in cigarette smoke models of lung injury [30].

Gene disruption of caveolin-1, which is known to be involved in integrin clustering and activation, results in pulmonary fibrosis and impairment in liver regeneration after partial hepatectomy which was reversible by treatment with glucose [31], indicating the probable importance of energy preservation. Interestingly, previous reports have shown that *β*1 integrin-mediated adhesion regulates cholesterol-rich membrane microdomain internalisation mediated by phosphocaveolin-1 [32] and caveolar endocytosis can be blocked by small interfering RNA knockdown of *β*1 integrin [33]. Our finding that allosteric modulation inhibits elastase-induced caveolin phosphorylation reinforces the idea that, in injury, abnormal integrin activation and clustering contribute to cellular damage in elastase-induced injury.

Integrin activation can occur via outside-inside and/or inside-outside signalling. We postulate from our results that outside-inside *β*1 integrin signalling and activation are induced during injury, possibly as a result of extracellular matrix degradation (reviewed in [1]). Matrix integrity has been shown to play a key role in various injuries including emphysema [34] and amyloid *β* neurotoxicity [35]. Indeed, unpublished data from our laboratory have shown that *β*1 integrin allosteric modulation using JB1a, but not 6S6 or TS2/16, caused an increase in perlecan [36]; a change partially sensitive to pretreatment with cycloheximide and the nonspecific metalloproteinase (MMPs) activator aminophenylmercuric acetate (APMA). The changes in perlecan in response to JB1a were accompanied by an increase in tissue inhibitors of metalloproteinase-1 (TIMP1) initially and pro-MMP-9 subsequently.

Integrin activation can also occur due to cellular changes [37, 38]. Various reports highlighted the effect of plasma membrane lipid composition on *β*1 integrin function [29, 39, 40]. Ceramide increase during elastase-induced injury has been shown to cause apoptosis [30, 41]. We have shown that, upon the onset of elastase-induced injury, neutral sphingomyelinase increased which may have contributed to *β*1 integrin activation; an effect inhibited by modulation of *β*1 integrin using JB1a.

Integrin activation is associated with increased engagement with the actin cytoskeleton [42, 43]. More recently, actin polymerisation has been shown to be affected in cigarette smoke models [44]. We have investigated actin polymerisation in our *in vitro* system during the course of elastase-induced injury and the effect of *β*1 integrin modulation on the process. We used live cell imaging of labelled monomeric actin incorporation to ascertain de novo increase in the formation of actin aggregates since the phalloidin staining fails to demonstrate the newly formed aggregate. We were able to show an increase in actin aggregates during the course of elastase-induced injury. This effect was inhibited by modulation of *β*1 integrin.

We then investigated how elastase-induced injury impacted on ATP. Under ATP depletion conditions, there is a net conversion of monomeric G-actin to polymeric F-actin resulting from an alteration in the ratio of ATP-G-actin and ADP-G-actin with the resultant F-actin forming dispersed aggregates [45]. We chose to characterise ATP dynamic changes *in vitro* following elastase-induced injury. We found not only the levels were reduced after prolonged exposure but preceding this reduction, abnormal fluctuations were detected at the onset of exposure to elastase. These responses were inhibited by allosteric modulation of *β*1 integrin.

With changes in cell membrane composition and actin cytoskeleton, we sought to confirm if those changes have impacted on cellular mechanical properties. We have shown that cellular impedance is altered during the course of elastase-induced injury and this effect was inhibited by modulation of *β*1 integrin. Although this measurement does not distinguish between effects caused by changes in cellular composition and cell-cell interaction, when taken together with evidence of alteration in actin polymerisation and cell membrane composition, it further supports our notion. There is strong evidence for the role of the state of the actin cytoskeleton on cell survival and differentiation which mainly came from studies focused on thymosin *β*4. Thymosin *β*4 functions mainly as a sequestering protein of actin monomers and promotes wound healing and cardiac repair by affecting cell survival [46].

Furthermore, we were able to show caspase activation in both end-point assays and real time. Elastase-induced caspase activation was inhibited by the modulation of *β*1 integrin. However, complete inhibition of *β*1 integrin using 6S6 clone (potent inhibitor and inducer of homotypic aggregation) has activated caspase.

Our findings support the hypothesis that cellular mechanics play a key part in cell fate and therefore affect repair. To investigate this in a disease setting in which remodelling is a key component, we established a murine model of emphysema caused by intratracheal instillation of elastase. Emphysema is an irreversible component of chronic obstructive pulmonary disease, a major cause of morbidity and mortality worldwide.

We hypothesised that, in irreversible moderately severe emphysema, *β*1 integrin becomes allosterically activated, with the corollary that only then might allosteric modulation become therapeutically beneficial. The expression of activation epitopes of *β*1 integrin, hence a fully extended active conformation, in human disease is poorly understood due to the technical limitations. However, recently, the presence of ligand competent *β*1 integrin in eosinophils from induced sputum samples of asthmatic patients was investigated and found to correlate with airway hyperresponsiveness [47]. Modulation of *β*1 integrin using JB1a reversed elastase-induced emphysema when administered at the two different time points after the onset and stabilisation of emphysema. It had no effect on vehicle instilled animals. This was confirmed by both unconscious lung function testing and structural analyses using the mean linear intercept. We have also previously determined that modulating *β*1 integrin function allows septation to proceed in damaged lungs by altering the pool of GATA-6 and TTF-1 expressing cells [48]. 

Although, the clone B44, which had the closest effect to JB1a-induced conformational effect, showed some effect on elastase-induced lung function abnormalities, it has decreased lung compliance in normal animals. We have not yet determined whether the clone B44 has induced fibrosis or alteration in airway responsiveness to account for the observed functional effects. 

Thus targeting *β*1 integrin with JB1a induced previously unseen disease-modifying effects. The key event, evident from our research, is the synergistic alteration of cell surface receptor distribution leading to alteration in receptor activity state and changes in the cell membrane composition. This effect makes cells more adaptable to their altered mechanical environment, thereby reducing the tendency of injury to cause increased cell stiffness, loss of energy, and ultimately death.

## 4. Material and Methods

### 4.1. Signalling

Adult human lung fibroblasts (ATCC, CCD-8Lu) were seeded onto collagen-I-coated BioFlex 6-well plates at 0.5 × 10^6^/well. The following day, NCI-H441 cells were seeded on top of the fibroblasts at the same density. Cells were starved with media containing 0.1% FCS. The plates were subjected to stretching at 2–10% sinusoidal stretch at 1 Hz for 2, 4, or 6 hours. PPE was added at 0.3 U/mL alone or in combination with JB1a (1 *μ*g/mL, gift from John Wilkins, Manitoba), AIIB2 (1 *μ*g/mL, Developmental Studies Hybridoma Bank of University of Iowa), or K20 (1 *μ*g/mL, Santa Cruz). At the end of the stretch, the media was aspirated and protein extracted from the cell layer using Bio-Plex cell lysis kit (Bio-Rad). The protein concentration in the lysates was measured using BCA method. Lysates were analysed for phosphoproteins (50 *μ*g/sample) using Bio-Plex Phospho 15-Plex assay kit (Bio-Rad) for Akt, c-Jun, CREB, ERK1/2, GSK3, histone H3, HSP27, I*κ*B, IRS-1, JNK, MEK1, P38 MAPK, Src, and STAT3 and 6. Measurements were according to manufacturer instructions.

### 4.2. *β*1 Integrin Imaging

Adult human lung fibroblasts (CCD-8Lu, ATCC, Rockville, MD) were seeded onto collagen-I-coated glass coverslips. The following day, NCI-H441 (ATCC, Rockville, MD) was seeded on top of the fibroblasts at the same density. Cells were starved with media containing 0.1% FCS. The plates were subjected PPE at 0.3 U/mL alone or in combination with JB1a (1 *μ*g/mL) or K20 (1 *μ*g/mL) for 1 and 3 hours. The cells were then fixed using ice-cold 4% paraformaldehyde. The cells were blocked using SuperBlock (Pierce) and double immunostained using antibodies against ligand competent *β*1 integrin (9EG7, BD Biosciences) and JB1a followed by Alexa 488 anti-rat and Alexa 555-anti-mouse, respectively, and nuclear staining with TO-PRO3. Images were acquired using Zeiss LSM 510 using ×40 oil lens and raw images presented using LSM image browser.

### 4.3. Fluorescence Resonance Energy Transfer (FRET)

The human leukemia Jurkat (clone E6-1) cell line was purchased from ATCC (Rockville, MD). Octadecyl rhodamine B chloride (R18) was from molecular probes. The FITC-conjugated analog of *α*4 specific peptide 4-((n′-2-methylphenyl)ureido)-phenylacetyl-L-leucyl-L-aspartyl-L-valyl-L-prolyl-L-alanyl-L-alanyl-L-lysine (LDV-FITC) was synthesized at Commonwealth Biotechnologies (Richmond, VA).

Cell- and bead-based fluorescence measurements were performed using BD LSRFortessa. The detailed analysis of LDV-FITC binding was described previously [20]. Cells were treated with a range of concentrations of the fluorescent ligand (typically 0–12 nM) in the presence of divalent cations (1 mM Mn^2+^), eventually choosing 4 nM for experiments. Similar studies were done for R18 and 10 um concentration achieved saturable binding. All experiments were performed in HEPES buffer (110 mM NaCl, 10 mM KCI, 10 mM glucose, 1 mM MgCl_2_, and 30 mM HEPES, pH 7.4) containing 0.1% FCA. Jurkat cells were used at a density of 1 × 10^6^ cells/mL. Kinetic analysis was done as described previously [20]. Briefly, cells were preincubated in HEPES buffer with or without divalent cations for 10 min at 37°C. Samples were analyzed for 30 s to establish a baseline, then the fluorescent ligand LDV-FITC was added and FACS ad. Additional measurements were carried out in the presence of anti-*β*1 integrin antibodies at 1–10 *μ*g/mL. Studies were done where the antibody was added 1 minute before the commencement of the measurements or 30 seconds after the addition of LDV-FITC without any difference observed. Data were acquired up 600 seconds to a total of 200,000 events. The data were converted to mean channel fluorescence over time using FlowJo software (Tree Star, Inc., Oregon, USA).

### 4.4. Cell Fractionation

#### 4.4.1. *β*1 Integrin

Adult human lung fibroblasts (CCD-8Lu) were seeded onto collagen-I-coated culture dishes. The following day, NCI-H441 cells were seeded on top of the fibroblasts at the same density. Cells were starved with media containing 0.1% FCS. The plates were subjected PPE at 0.3 U/mL alone or in combination with JB1a (1 *μ*g/mL) or K20 (1 *μ*g/mL) for 1 and 3 hours. At the end of the experiment, media was aspirated and cell layer extracted using MEM-PER protein extraction kit (Pierce), protein assayed in the membrane fraction using the BCA methods, and 50 *μ*g separated onto 10% SDS-PAGE, transferred onto Hybond-ECL (GE Healthcare). All membranes were stained with Ponceau S (Sigma) to assess the quality of the transfer and loading and probed for *β*1 integrin (JB1a) followed by HRP-labelled secondary antibody and developed using ECL-Plus (GE Healthcare) and exposed to Hyperfilm ECL (GE Healthcare). Densitometric analyses was carried out using ImageJ (NIH).

#### 4.4.2. Caveolin

In an additional set of experiments, cells were cultured as described onto collagen-I-coated Bioflex plates and subjected to stretch at 2–10% for 10, 30 minutes, 1 or 3 hours. PPE was added at 0.3 U/mL alone or in combination with PPE was added at 0.3 U/mL alone or in combination with JB1a (1 *μ*g/mL), AIIB2 (1 *μ*g/mL), or K20 (1 *μ*g/mL). At the end of the stretch, the media was aspirated, and protein extracted from the cell layer was fractionated using the compartmental protein extraction kit (CNMCS, Biochain). Protein content was measured using the BCA methods. Lysates were separated onto 10% SDS-PAGE using Biorad's Mini Protean 3 Dodeca electrophoresis cell which allows running 12 gels simultaneously to ensure validity for densitometric analyses. The gels were transferred onto ECL-hybond ECL. All membranes were stained with Ponceau S (Sigma) to assess the quality of the transfer and loading. ECL membranes were probed for *β*1 integrin using JB1a (generous gift from John Wilkins, Manitoba) and mouse actin antibody (NH3, Abcam), or caveolin (rabbit anti-human, BD Biosciences) and phosphocaveolin 1 (BD Biosciences). Secondary detection was done using 680 nm and 800 nm fluorescent antibodies (LiCor), and images of the blots were acquired using LiCor system. Densitometric analyses was carried out using ImageJ (NIH).

#### 4.4.3. Sphingomyelinase Activity

Adult human lung fibroblasts (CCD-8Lu) were seeded onto collagen-I-coated BioFlex 6 well plates at 0.5 × 10^6^/well. The following day, NCI-H441 cells were seeded on top of the fibroblasts at the same density. Cells were starved with media containing 0.1% FCS. The plates were subjected to stretching at 2–10% sinusoidal stretch at 1 Hz for 2, 4, or 6 hours. PPE was added at 0.3 U/mL alone or in combination with JB1a (1 *μ*g/mL). At the end of the stretch, the media was aspirated, snap frozen in liquid nitrogen, and lyophilised and enzyme activities assayed using Amplex red sphingomyelinase assay kit (Invitrogen) according to the manufacturers' instructions.

#### 4.4.4. Time-Lapse Studies

Cells were cultured as described in methods at 50,000 cell/membrane onto collagen I Bioflex membranes using silicone gaskets of 10 mm diameter. Cells were starved with media containing 0.1% FCS and Syto 16 (Molecular Probes). The media was removed, and Alexa-Fluor 647 labelled G-actin (100 *μ*g/membrane) from rabbit was loaded using Influx (Molecular Probes). The cells were loaded with PhiPhiLux-G_2_D_2_ for visualisation of caspase activation (OncoImmune). The membrane was then mounted onto the StageFlexer (FlexCell), placed on the stage of an upright Leica-TCS-NT confocal microscope system (Leica Microsystems GmbH, Heidelberg, Germany), and subjected to 2–10% cyclic stretch at 1 Hz for up to 6 hours. Images were collected simultaneously from 3 channels at 1-minute intervals, using the ×10 lens. The resulting time-lapse movies were collated and analysed with Imaris software (Bitplane AG, Switzerland). At various time points during the study, the membrane was held static while serial optical sections were acquired the three fluorescent channels supplemented by the collection of the brightfield channel image.

#### 4.4.5. Three-Dimensional Confocal Microscopy

NCI-H441 cells and human lung fibroblasts were cultured as described above onto collagen-I-coated glass coverslips at 20,000 cells within an area of 5 mm in diameter. The media was removed and Alexa-Fluor 647 labelled G-actin (30 *μ*g/coverslip) from rabbit was loaded using Influx (Molecular Probes). The cells were loaded with PhiPhiLux-G_2_D_2_ for visualisation of caspase activation (OncoImmune) and FL-ganglioside 1 (GM1, Molecular probes) to visualise the plasma membrane. Images were collected through 4 separate channels (GM1: *λ* = 488, caspase *λ* = 568, actin: *λ* = 647 and brightfield) using x63 water lens and Zeiss LSM510 CLSM microscope. The resulting images were analysed with Imaris software (Bitplane AG, Switzerland). Three-dimensional images were reconstructed.

#### 4.4.6. ATP Measurments

In a separate set of experiments lung fibroblasts and epithelial cells were seeded onto 96 multi-well plates as described above. The cells were starved in media containing 0.1% FCS then in DMEM-glucose-free with 0.1% FCS for 45 minutes before treating with (i) PPE at 0.3 U/mL alone or (ii) PPE preceded by JB1a (1 *μ*g/mL). At the end of the experiment, ATP levels were measured using a bioluminescent ATP kit (Perkin Elmer).

#### 4.4.7. Electrical Impedance

ECIS monitors the impedance of small 250-micrometer diameter electrodes used as substrates for cell growth. When cells grow on the electrode, they impede current flow. Cells were layered as described before onto slides (8W10E, Applied Biophysics) which contain 8 wells each containing ten circular 250 *μ*m diameter active electrodes connected in parallel on a common gold pad. PPE was added at 0.3 U/mL alone or in combination with JB1a (1 *μ*g/mL). Impedance was monitored using ECIS controller model 1600 (Applied Biophysics).

### 4.5. Caspase Activation Measurements in Human Mesenchymal and Epithelial Cell Coculture

Adult human lung fibroblasts (CCD-8Lu) were seeded onto collagen-I-coated BioFlex 6-well plates at 0.5 × 10^6^/well. The following day, NCI-H441 cells were seeded on top of the fibroblasts at the same density. Cells were starved with media containing 0.1% FCS. The plates were subjected to stretching at 0–5%, 0–10%, or 2–10% sinusoidal stretch at 1 Hz for 6 hours. Control plates on plastic or bioflex plates without stretch were also included. PPE was added at 0.3 U/mL alone or in combination with JB1a (1 *μ*g/mL), 6S6 (1 mg/mL), or ZVAD-fmk at 10 *μ*M. At the end of exposure period which was 1, 3, and 6 hours, the media was aspirated and caspase 3 activity assayed using Caspase-Glo 3/7 (Promega) according to the manufacturers' instructions.

### 4.6. Cross-Reactivity of *β*1 Integrin Antibodies

Human and murine neuronal cells were fixed in formaldehyde. The cells were blocked using SuperBlock (Pierce) and immunostained using antibodies against *β*1 integrin (JB1a and B44, gift from John Wilkins, Manitoba) followed by Alexa 647 anti-mouse (Molecular Probes) and nuclear staining with 7-AAD (Molecular Probes). Image z stacks were acquired using Zeiss LSM 510 with Nyquist calculator settings (http://www.svi.nl/NyquistCalculator) using Plan-Neofluar 40x/1.30 Oil DIC lens and raw images deconuolved and presented in 3d using Huygens software (Scientific Volume Imaging SVI, The Netherlands).

### 4.7. PPE-Induced Air Space Enlargement Model in Mice

Female C57/BL6 mice (6–8 weeks old) were instilled intratracheally with porcine pancreatic elastase (Roche) as detailed before [49] at 0.2 U/g. All procedures were approved by the Ethical Review Committee of the University of Edinburgh. The procedures were carried out under a project licence number PPL60/3984 issued by the Home Office under the UK Animals (Scientific Procedures) Act 1996. At day 14 (21 d group) or 21 and 28 days (35 d group), mice were treated intratracheally with the anti-integrin antibody JB1a at 3 mg/kg in sterile PBS. The dose chosen is equivalent to the dose of clinically used antibodies against *α*4*β*1 integrin [50]. Control group was instilled initially with PBS with either JB1a or B44 at day 14 (21 d) or at days 21, and 28 (35 d). Additional vehicle control groups were instilled with PBS at days 1 and 14 (21 d) or days 1, 21 and 28 (35 d). We have carried out studies where control groups were instilled with an isotype control (MOPC21, Sigma-Aldrich) and no effect was seen. For the group treated at day 14, the animals were terminated at day 21 (21 d), and, for the group treated on days 21 and 28, the animals were terminated on day 35 (35 d) as follows. The animals were anaesthetised using sodium pentobarbitone (45 mg/kg), paralysed using pancuronium bromide (0.8 mg/kg), and tracheostomised and ventilated using a small animal ventilator (Flexivent, SCIREQ, Montreal) at 8 mL/kg and a rate of 150 breaths/minute and positive end expiratory pressures (PEEP) of 3 cmH_2_O. 

The pressure-volume curve was obtained during inflation and deflation in a stepwise manner by applying volume perturbation incrementally during 16 seconds. The pressure signal was recorded and the pressure-volume (P-V) curve calculated from the plateau of each step. The constant K was obtained using the Salazar-Knowles equation and reflects the curvature of the upper portion of the deflation P-V curve. Quasistatic elastance reflects the static elastic recoil pressure of the lungs at a given lung volume. It was obtained by calculating the slope of the linear part of P-V curve. 

In an additional study, female C57/BL6 mice (6–8 weeks old) were instilled intratracheally with 0.3 U/g porcine pancreatic elastase (Elastin products) as detailed above. At days 21 and 28, mice were treated intratracheally with either the anti-integrin antibody JB1a or B44 at 3 mg/kg in sterile PBS. Control group was instilled initially with PBS with either JB1a or B44 at days 21 and 28. An additional vehicle control group was instilled with PBS at days 1, 21, and 28. Lung function was assessed as described above.

### 4.8. Histochemistry

After the measurements, the animals were sacrificed and lungs were removed and formalin-fixed at a pressure of 25 cm H_2_O, paraffin-embedded, and sectioned at 4 *μ*m thickness. Sagittal sections were used from each animal for histological and immunohistochemical assessment of damage and morphometric analysis (mean linear intercept, Lm). Images from 10 fields per section were digitised using Image-Pro plus (version 5.1) and micropublisher 3.3 RTV camera connected to a Zeiss Axioskope with 10x objective. The field size was 0.83 *μ*m × 0.63 *μ*m. Mean linear intercept was calculated from each field (horizontal and vertical) by dividing the length of the line by the number of alveolar intercepts.

### 4.9. Apoptosis Measurement

Terminal deoxyribonucleotidyl transferase- (TdT-) mediated dUTP Nick End Labelling (TUNEL) was assessed in sections using the Red ApopTagTM Kit (Chemicon). Data for the quantification of positively stained apoptotic nuclei was acquired using the ×40 oil objective of a Zeiss 510 Axiovert confocal microscope system (Carl Zeiss Ltd, Welwyn Gardens City, Herts, UK). The stage-tiling utility was employed for the collection of 4 × 4 tiled images, equivalent to a total area of 0.921 mm × 0.921 mm, imaged from a lung section of ~8 mm × 8 mm (two tiles each from right and left lobes). Images of mainly alveolar tissue were constructed. The images were then converted to 8-bits grey scale, and Image J was used to count total number of cells. TUNEL positive cells were counted manually.

### 4.10. Statistical Analyses

All data were analysed using SPSS for windows. Data were analysed using the general linear model and multivariate ANOVA with post hoc *t*-test.

## Supplementary Material

Time lapse video of epithelial-mesenchymal cultures during stretch at 2–10% amplitude at 1 Hz for up to 6 hours (compressed videos) demonstrating the S1. Baseline formation of F-actin (blue) and caspase 3/7 activation (red, Sytox green was used for cell tracking) in control cultures, S2. The increase in formation of F-actin (blue) and caspase 3/7 activation (red) in response to elastase (PPE, 0.6 U/mL), and S3. The inhibition of the increase in F-actin (blue) and caspase 3/7 activation (red) in response to elastase (PPE, 0.6 U/mL) by targeting *β*1 integrin using JB1a (1 ug/mL).
